# Dysregulation of Na+/K+ ATPase by amyloid in APP+PS1 transgenic mice

**DOI:** 10.1186/1471-2202-6-7

**Published:** 2005-02-02

**Authors:** Chad A Dickey, Marcia N Gordon, Donna M Wilcock, Donna L Herber, Melissa J Freeman, Dave Morgan

**Affiliations:** 1 Alzheimer's Disease Research Laboratory, Department of Pharmacology, University of South Florida, Tampa, USA

## Abstract

**Background:**

The pathology of Alzheimer's disease (AD) is comprised of extracellular amyloid plaques, intracellular tau tangles, dystrophic neurites and neurodegeneration. The mechanisms by which these various pathological features arise are under intense investigation. Here, expanding upon pilot gene expression studies, we have further analyzed the relationship between Na+/K+ ATPase and amyloid using APP+PS1 transgenic mice, a model that develops amyloid plaques and memory deficits in the absence of tangle formation and neuronal or synaptic loss.

**Results:**

We report that in addition to decreased mRNA expression, there was decreased overall Na+/K+ ATPase enzyme activity in the amyloid-containing hippocampi of the APP+PS1 mice (although not in the amyloid-free cerebellum). In addition, dual immunolabeling revealed an absence of Na+/K+ ATPase staining in a zone surrounding congophilic plaques that was occupied by dystrophic neurites. We also demonstrate that cerebral Na+/K+ ATPase activity can be directly inhibited by high concentrations of soluble Aβ.

**Conclusions:**

The data suggest that the reductions in Na+/K+ ATPase activity in Alzheimer tissue may not be purely secondary to neuronal loss, but may results from direct effects of amyloid on this enzyme. This disruption of ion homeostasis and osmotic balance may interfere with normal electrotonic properties of dendrites, blocking intraneuronal signal processing, and contribute to neuritic dystrophia. These results suggest that therapies aimed at enhancing Na+/K+ ATPase activity in AD may improve symptoms and/or delay disease progression.

## Background

Alzheimer's disease (AD) has several well-characterized post-mortem pathological markers that include both gliosis and dystrophic neurites surrounding extracellular amyloid plaques. In addition, intracellular tangles of hyper-phosphorylated tau and massive neurodegeneration are seen later in the disease process. Mutated forms of both amyloid precursor protein (APP) and presenilin 1 (PS1) lead to an increased rate of amyloid deposition and therefore an earlier onset of the dementia associated with AD [1]. Doubly transgenic mice expressing these human mutants of the APP [2] and PS1 [3] genes (APP+PS1 mice; [4] exhibit a large amount of amyloid deposition and gliosis without the formation of tangles or neuron loss, and yet they still develop anterograde amnesia as they age, similar to what is seen in the early stages of AD [5]. Memory deficits without the loss of neurons indicate that amyloid-associated disruption of some step in neural processing can result in memory deficits.

Previously we have described decreased expression of genes critical for learning and memory and impaired induction of several immediate early genes (IEGs) in aged, memory deficient APP+PS1 mice [6,7]. Increased neural activity during learning is argued to be a primary inducing stimulus for these IEGs [8]. One possible mechanism to describe this phenomenon would be that amyloid is diminishing the ability of neurons to facilitate sufficient electrical signaling to induce changes in synaptic plasticity essential for memory consolidation. Here we present evidence that in addition to decreased expression of Na+/K+ ATPase mRNA as previously described [6,7], the activity of this enzyme is significantly decreased in the APP+PS1 hippocampus but not in the amyloid-free cerebellum. Perhaps not surprisingly ouabain, an Na+/K+ ATPase inhibitor, has been shown to impair memory consolidation [9,10]. We decided to investigate further the interactions of Aβ and Na+/K+ ATPase activity to understand better the potential role of this enzyme in AD and memory dysfunction.

## Results

We used the APP+PS1 transgenic mouse model to better understand how the deposition of amyloid contributes to the consistent memory loss seen in these animals [11-14]. Specifically, we have found that Na+/K+ ATPase, a protein critical for not only brain function, but survival, is adversely affected by the presence of amyloid and this interaction may be driving a major pathological event in the progression of amyloid-associated dementia.

**Figure 1:**
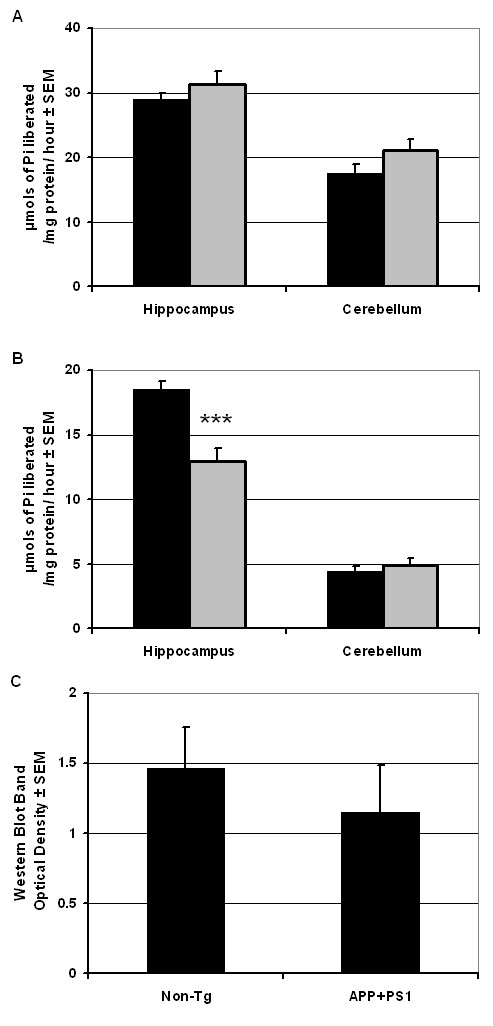
*Reduced activity and protein levels of the Na+/K+ ATPase enzyme in APP+PS1 mice. *Activity of total (A) and ouabain-sensitive (B) ATPase was assayed colorimetrically in APP+PS1 mice tissue (n = 8, open bars) and non-transgenic littermate tissue (n = 8, solid bars), presented here as μmols of phosphate liberated by ATPase per mg of protein per hour. In the amyloid-containing hippocampus, there was a significant decrease in the specific activity of ouabain-sensitive ATPase in APP+PS1 mice compared to non-transgenic mice, but there was no decrease in cerebellar Na+/K+ ATPase activity. Cerebellar activity was 20% of that seen in non-transgenic hippocampus. Panel C shows the quantitation of the optical density of bands corresponding to the molecular weight of Na+/K+ ATPase using standard Western blot technique. This reveals a trend for decreased protein levels. *** indicates significant differences between APP+PS1 mice and non-transgenic littermates (p < 0.001) when measured by one-way ANOVA.

In order to determine if our early observation regarding reduced mRNA for Na+/K+ ATPase was functionally meaningful, we measured total and ouabain-sensitive ATPase activity in APP+PS1 brain homogenates compared to those from non-transgenic littermates using a standard colorimetric assay. Using ouabain as a selective inhibitor of Na+/K+ ATPase, we demonstrated that the specific enzymatic activity was significantly reduced by ~25% in the hippocampi of aged, memory-deficient APP+PS1 mice compared with non-transgenic littermates (Figure 1B). The percent reduction in total ATPase activity was similar to that of specific Na+/K+ ATPase activity, suggesting that inhibition of is selective for Na+/K+ ATPase (Figure 1A &1B). In addition, approximately 40% of all the ATP hydrolysis found in the homogenate was ouabain sensitive, confirming that this enzyme is likely the largest single site of ATP hydrolysis in brain. The specific activity values measured here were consistent with those reported 3 decades ago by Stefanovic et al. [15], testifying to the robustness of the assay method. Upon analysis of the cerebellum of these mice, we found that the specific enzyme activity in APP+PS1 transgenics remained equivalent to that in non-transgenic littermates (Figure 1B); however the overall activity of Na+/K+ ATPase in this region was only one fifth of that in the hippocampus (Figure 1A &1B). Western blot analyses of cortical homogenates demonstrated a trend for reduced protein expression, a finding that would be expected based upon the mRNA analyses (Figure 1C).

**Figure 2:**
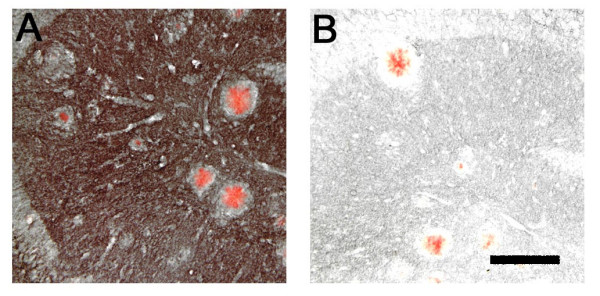
*Verification of Na+/K+ ATPase αIII antibody specificity by immunohistochemistry following pre-incubation with purified protein and Western blotting*. Horizontal sections were immunostained for Na+/K+ ATPase αIII with (Panel B) and without (Panel A) pre-incubation with 70 μunits of purified enzyme followed by Congo red staining. The pre-incubation significantly decreased staining, confirming antibody specificity. Scale bar = 50 μm.

**Figure 3:**
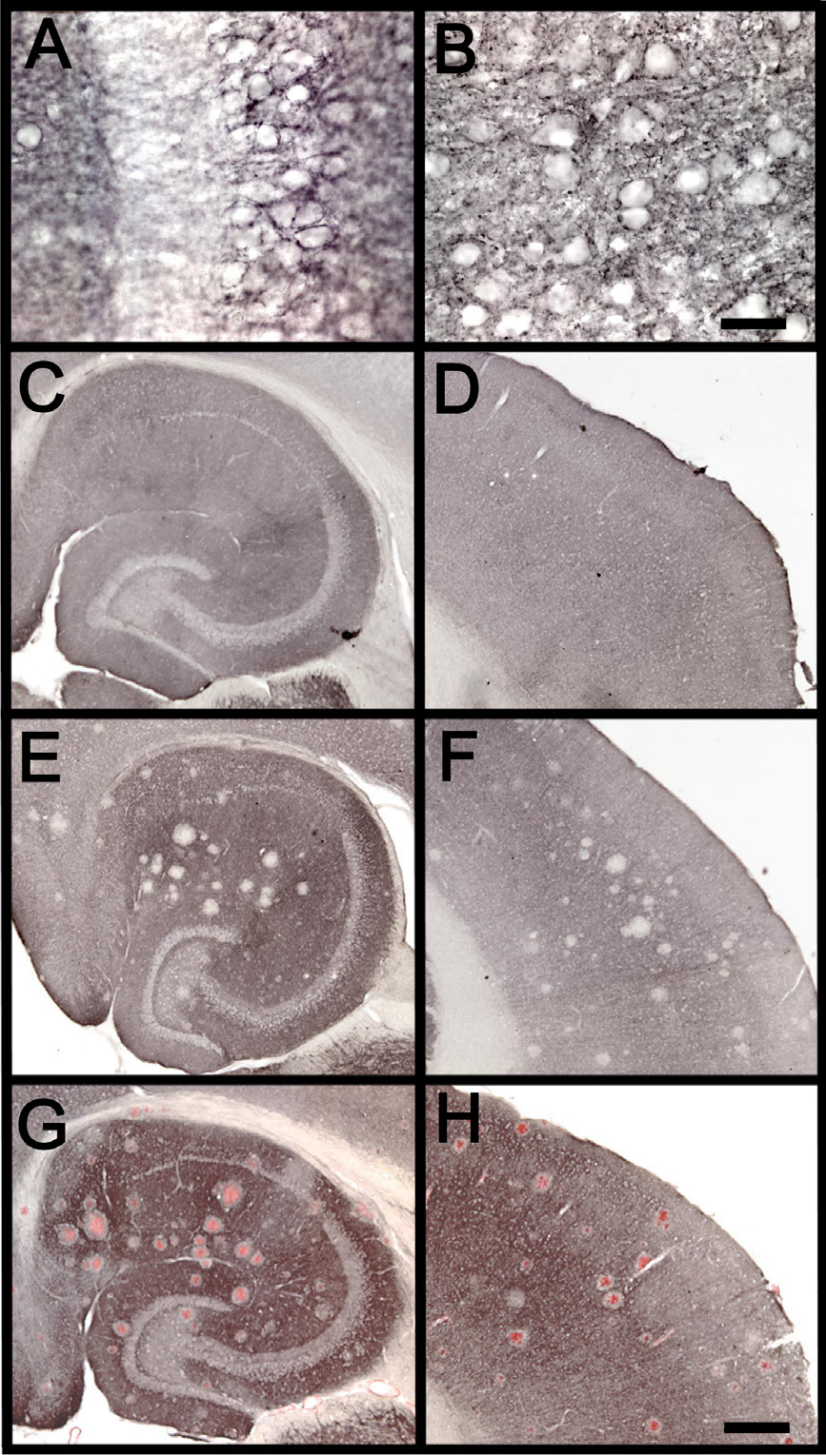
*Hippocampal and cortical immunohistochemistry for Na+/K+ ATPase in APP+PS1 mice and non-transgenic littermates*. Horizontal hippocampal and cortical sections were immunostained for Na+/K+ ATPase αII. The left panels depict hippocampus, while the right panels encompass the cerebral cortex. Panels A & B: high power magnification (Scale bar = 8.33 μm) revealed membrane-localized staining for Na+/K+ ATPase in the insular cortex (Panel B) and CA3 of the hippocampus (Panel A) of non-transgenic mice. Panel C: hippocampal staining of non-transgenic mice showed a ubiquitous distribution of Na+/K+ ATPase throughout the neuropil, while less staining was apparent along the pyramidal layer of Ammon's horn, the hilus and the granular layer of the dentate gyrus. Panel D: cortical staining was also substantial with slight lightening in layers 1 and 2. White matter in both structures remained unstained. A similar staining pattern was observed in the APP+PS1 mice with the exception of focal non-stained areas in the gray matter, presumably where amyloid plaques are located (Panel E [hippocampus] & F [cortex]). Immunostaining followed by Congo red histochemistry confirmed that the Na+/K+ ATPase staining was absent not only immediately where amyloid was located, but also in a zone surrounding the plaque (Panels G [hippocampus] & H [cortex]). Scale bar for panels C-H = 120 μm.

Immunohistochemical staining for the Na+/K+ ATPase αIII subunit was performed using saggital sections from non-transgenic and APP+PS1 mice. Specific staining was observed throughout the forebrain. The specificity of the Na+/K+ ATPase α subunit antibody used for immunochemistry was determined by pre-incubation of the antibody with purified Na+/K+ ATPase protein, which dramatically reduced apparent immunostaining (Figure 2B) compared with normal staining (Figure 2A; amyloid plaques stained with Congo red dye appear red). Positive reaction product appeared to be localized to the periphery of cellular profiles in CA3 of the hippocampus (Figure 3A) and the insular cortex (Figure 3B), consistent with the enzyme's membrane-association. Throughout the brain, the white matter regions appeared to have less intense staining, than the neuropil, consistent with the larger numbers of ions crossing the membrane in post-synaptic potentials than action potentials (thus requiring more ionic pumping to maintain ionic equilibrium). Additionally, regions with high densities of neuronal somata also appeared to have less dense staining, reflecting the greater density of membrane area in dendritic than somatic regions of neuropil. Similar observations have been made using [3H] ouabain to mark Na+/K+ ATPase distribution in brain [16] Another striking observation from images of the hippocampus in non-transgenic mice (Figure 3C) demonstrate a decreased intensity of staining along the dentate granule cell projection pathways within the hilus and along the mossy fiber projections to CA3 (Figure 3A). This can also be observed at higher magnification in figure 3A, where there is reduced staining to the left of the somatic staining in the inner molecular layer. Although not commented upon, this too can be observed in micrographs of [3H] ouabain autoradiography [17].

**Figure 4:**
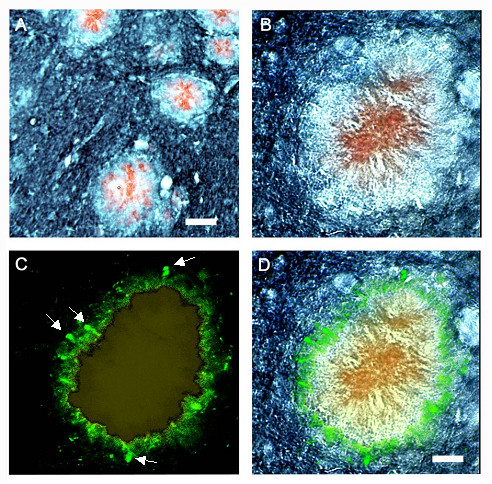
*Dual Immunostaining of Na+/K+ ATPase αIII and dystrophic neurites*. Horizontal sections were immunostained for Na+/K+ ATPase using DAB with nickel intensification followed by Congo red staining for Aβ plaques. A discernable circumferential void in ATPase staining surrounding the plaque was observed (Panel A, scale bar = 50 μm; Panel B, scale bar = 16.67 μm). Immunofluorescent staining of dystrophic neurites using the anti-phosphorylated neurofilament antibody SMI-312 followed by Congo red staining demonstrate a close relationship between amyloid plaques and dystrophic neurites (Panel C; white arrows demarcate the neurites). Congo red yellow fluorescence was digitally suppressed to more clearly reveal the green-stained neurites (scale bar = 16.67 μm). Panel D is a digital overlay of the fluorescein-labeled dystrophic neurite image onto the bright field peroxidase-labeled Na+/K+ ATPase + Congo red image which demonstrates that dystrophic neurites are present predominantly in the zone devoid of Na+/K+ ATPase staining surrounding the congophilic plaques (scale bar = 16.67 μm).

Cortical staining (Figure 3D) revealed a fairly uniform pattern, with the exclusion of white matter. The difference in Na+/K+ ATPase staining between non-transgenic and APP+PS1 forebrain areas is the absence of staining where amyloid plaques are present in the APP+PS1 mice (Figure 3E &3F). Subsequent Congo red staining of these amyloid plaques confirmed that the loss of Na+/K+ ATPase staining was not only present in the center of the plaque, but also in a penumbral zone immediately surrounding the congophilic material, resulting in a "halo" (Figure 2A, 3G, 3H &4A). Higher power magnification clearly demonstrated the lack of Na+/K+ ATPase staining surrounding the congophilic plaques (Figure 4A and 4B).

These findings led us to postulate that the osmotic imbalance brought on by an absence or inhibition of Na+/K+ ATPase may contribute to the swelling associated with dystrophic neurites found in the vicinity of congophilic plaques in APP+PS1 mice and AD patients. We designed a dual immunostain utilizing immunohistochemical methods for Na+/K+ ATPase and immunofluorescence methods for phosphorylated neurofilament, a dystrophic neurite marker we evaluated earlier [12]. Using this assay, we were able to demonstrate that the dystrophic neurites were almost exclusively present within the zone surrounding the congophilic plaque that lacked Na+/K+ ATPase staining. This is represented in a triple staining overlay of the fluorescent neurites from figure 4C onto the bright field image of Na+/K+ ATPase and Congo red staining from figure 4B (Figure 4D). The arrows in figure 4C demarcate where dystrophic neurites are present.

**Figure 5:**
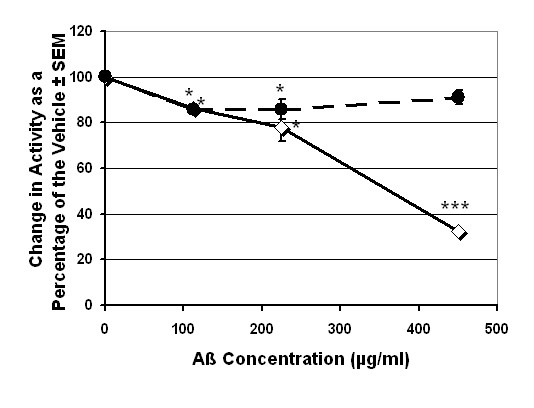
*Amyloid-beta 1–42 peptide inhibits Na+/K+ ATPase activity*. Purified cerebral Na+/K+ ATPase was pre-incubated with vehicle or Aβ 1–42, and enzyme activity percentages of the vehicle (20 μmols Pi/mg protein/hour) are presented (mean ± SEM). "◊" indicates the values for the soluble (DMSO+water) Aβ suspension. "○" indicates the values for the fibrillar (DMSO+neutralized HCl) Aβ suspension. At the highest concentrations, the soluble preparation dramatically reduces Na+/K+ ATPase activity compared to both vehicle and the fibrillar Aβ preparation. * (p < 0.05) and *** (p < 0.001) indicates significant differences between Aβ and vehicle alone when measured by one-way ANOVA.

Finally, to determine whether amyloid could inactivate Na+/K+ ATPase activity, we measured the activity of purified cerebral Na+/K+ ATPase after exposure to various concentrations of Aβ 1–42 peptide in a DMSO+water suspension or a DMSO+neutralized HCl suspension. Figure 5 demonstrates that increasing concentrations of the DMSO+water Aβ soluble preparation dose-dependently reduced Na+/K+ ATPase activity, whereas the fibrillar Aβ preparation suspended in DMSO+neutralized HCl did not demonstrate the same effect. There were significant reductions in Na+/K+ ATPase activity at the lower concentrations of 112 and 225 μg/ml compared to vehicle, but maximal reductions were at the highest concentration of the DMSO+water Aβ suspension (450 μg/ml).

## Discussion

Over the past 6 years, our group has characterized various aspects of the APP+PS1 transgenic mice including their pathology, behavior, and gene expression. With age, these mice progressively develop more amyloid plaques surrounded by dystrophic neurites, activated microglia and astrocytes [12]. With increasing amyloid burden, aged animals consistently develop memory deficits in the radial arm water maze (RAWM; [13,18,19], and, there is as yet no evidence for neuronal or synaptic loss. [6,20,21]. We have also demonstrated that several genes critical for synaptic plasticity and memory consolidation are down-regulated in these mice exclusively in those brain regions which accumulate amyloid [6] and the induction of a subset of immediate-early genes is impaired when the transgenic mice are introduced to a novel environment [7].

Here, we show that Na+/K+ ATPase has decreased enzyme activity in the amyloid-containing hippocampus of APP+PS1 transgenic mice. We have also demonstrated by immunohistology that Na+/K+ ATPase protein expression is reduced in the immediate vicinity of congophilic plaques, a zone where dystrophic neurites are most prevalent, suggesting that disrupted ionic homeostasis may contribute to their formation. Additionally, high concentrations of Aβ 1–42 directly inhibit the activity of Na+/K+ ATPase. This suggests that in the area surrounding amyloid plaques, where the local Aβ concentration is likely high, Na+/K+ ATPase activity may be locally inhibited.

From previous gene expression studies, we found that the mRNA for the Na+/K+ ATPase αIII subunit was consistently down-regulated ~30% in the hippocampi of APP+PS1 mice compared to non-transgenic littermates and to the amyloid-free cerebella [6]. These reductions were also demonstrated in human Alzheimer's disease samples, consistent with data from previous investigations [6,22]. Using a sensitive colorimetric assay to measure activity of Na+/K+ ATPase modified from Ellis et al. [23], we were able to demonstrate that in the APP+PS1 hippocampus, the specific activity of ouabain-sensitive ATPase was significantly reduced (figure 1B) while Na+/K+ ATPase activity in the amyloid-free cerebellum remained unperturbed with respect to genotype. Cerebellar activity was substantially lower than that seen in the non-transgenic hippocampal tissue, perhaps indicative of the abundant white matter found in this region, where Na+/K+ ATPase activity is low. These data demonstrate that the function of Na+/K+ ATPase is perturbed in a brain region that contains high overall concentrations of Aβ.

Previous investigations have shown that Na+/K+ ATPase protein levels are decreased in AD tissue but not in normal aged tissue [24,25], but it is difficult to dissociate the loss due to neuronal death from any loss caused directly by Aβ inhibition. This demonstration that reduced activity along with a trend for reduced protein levels can be determined in homogenates from animal models of amyloid deposition argues that at least some of this loss in AD brain is associated with direct actions of Aβ. In vitro data suggests that Na+/K+ ATPase activity can be blocked directly by various Aβ peptide fragments in cultured neurons [26] and that even the purified enzyme can be inhibited by high concentrations of Aβ (Figure 5).

When we immunostained transgenic mouse tissue to visualize the distribution of Na+/K+ ATPase alpha III subunits we found that in areas where congophilic plaque staining was apparent, Na+/K+ ATPase staining was absent, and more specifically, there appeared to be no or little Na+/K+ ATPase staining in a penumbral zone surrounding the plaques stained with Congo red (Figures 3E–H and 4A–B). While most immunostaining protocols detect voids at the sites where congophilic plaques are located, these areas appeared somewhat larger in the Na+/K+ ATPase immunostained sections. This led us to speculate about that a reduction in the activity of this extremely important enzyme may have severe impacts on the functions of neurons in the vicinity of the deposits.

We knew from previous studies that dystrophic neurites could be visualized surrounding amyloid plaques in the APP+PS1 mice. And we have demonstrated previously that proteins such as synaptophysin and APP are in fact increased in dystrophic neurites [12]. Therefore, we decided to stain sections for Na+/K+ ATPase and dystrophic neurites, along with amyloid plaques using Congo red, to determine whether Na+/K+ ATPase was absent from these neurites and in their immediate vicinity. We found empirically that staining the ATPase with a peroxidase label and the phosphorylated neurofilament with a fluorescein label to detect dystrophic neurites was the most effective way to see both markers on the same section along with the Congo red stained plaques. Imaging of these sections revealed that dystrophic neurites are in the circumferential area surrounding the congophilic amyloid plaques where Na+/K+ ATPase staining is absent (Figure 4D).

One possible explanation for this putative relationship would be that Aβ associated inhibition of Na+/K+ ATPase activity would result in osmotic imbalance and cause the neurites to begin swelling. In addition to the loss of electronic properties and accompanying dysregulation of neuronal signaling, these changes might even feedback to influence gene expression. Indeed, Huang et al. demonstrated that cells exposed to ouabain have reduced Na+/K+ ATPase αIII subunit mRNA expression [27]. An alternative pathway may involve interactions of Aβ with surface proteins, such as integrins [28,29]. and focal adhesion proteins [30] leading to activation of signal transduction cascades that mediate tyrosine phosphorylation [31]. Bozulic et al. reported that the tyrosine kinase, Lyn, can phosphorylate Na+/K+ ATPase resulting in its removal from the membrane [32]. These findings suggest that either direct inhibition of Na+/K+ ATPase by amyloid or its removal due to amyloid-mediated activation of a signaling cascade, could contribute to the formation of dystrophic neurites due to osmotic and/or ionic imbalances.

Aβ has been shown to bind various cell surface proteins [26,33,34]. and induce neuro-toxicity *in vitro *[35-37]. To determine the effect of Aβ 1–42 on Na+/K+ ATPase activity, we pre-incubated purified Na+/K+ ATPase with Aβ then colorimetrically measured enzyme activity. Although both preparations of Aβ did suppress activity at 112 μg/ml (~10 μM) and 225 μg/ml (~50 μM), it was the highest concentration (450 μg/ml or ~100 μM) of the soluble Aβ suspension that precipitated the largest reduction in activity compared to vehicle, nearly rendering it completely inactive (Figure 5). The fibrillar Aβ preparation did not exact the same precipitous decline in activity, demonstrating that it is not simply an artifact caused by high concentrations of a peptide that reduced activity. Further investigations will be required to determine which physicochemical form of Aβ is causing the reduced activity. This suggests that the Aβ can directly bind to the Na+/K+ ATPase and decrease its activity. Mark et al. suggests that the 25–35 amino acid region of the Aβ peptide induces oxidative stress thereby impairing Na+/K+ ATPase activity [26], and the findings presented herein are consistent with this earlier work.

## Conclusions

These data indicate that Aβ deposition in transgenic mice is associated with reduced activity of Na+/K+ ATPase. In vitro studies suggest that high concentrations of Aβ can quickly inactivate the enzyme activity. One area in the brain that might harbor Aβ concentrations sufficient to suppress the activity of Na+/K+ ATPase would be the micro-domain near and immediately around the plaques. This is the area demonstrated to have reduced immunostaining for Na+/K+ ATPase, while exhibiting increased phosphorylated neurofilament staining consistent with dystrophic neurites. These results lead to the possibility that one factor contributing to the formation of dystrophic neurites is loss of ionic homeostasis. Such changes might explain the "swollen" nature of these neuronal processes in the vicinity of plaques. It might also lead to sufficient disruption of electro-chemical properties as to disrupt normal information processing and lead to memory dysfunction. If these suggestions are correct, drugs targeted at activating Na+/K+ ATPase and maintaining ionic balance in these neurons may benefit Alzheimer's patients by delaying the onset of neuritic dystrophia and memory dysfunction.

## Methods

### Tissue preparation

Mice were bred in our facility and genotyped using previously described methods [19] The working memory performance of the APP+PS1 mice used in these studies was impaired when compared to non-transgenic littermates as published previously [14]; untreated groups were studied here). For tissue collection, 17–18 month old mice were deeply anesthetized with pentobarbital (100 mg/kg) and perfused transcardially with phosphate buffered saline. Brains were removed and bisected into right and left hemispheres. The right hemisphere was immediately dissected into regions that were immediately frozen on dry ice, while the left hemisphere was post-fixed in 4% para-formaldehyde for 24 hours and subsequently processed through a cryo-protection schedule of 10, 20 and 30% sucrose. Frozen brains were sectioned horizontally on a sliding microtome at 25 μm and stored in Dulbecco's phosphate buffered saline plus azide at 4°C.

### Na+/K+ ATPase activity assay and amyloid preparation

An assay to detect specific activity of Na+/K+ ATPase by measuring the release of phosphate was developed using a variation of the method described by Ellis et al. [23]. Freshly frozen dissected hippocampi, cortex and cerebella (20–30 mg tissue weight) from APP+PS1 and non-transgenic littermates were homogenized using a rotor-stator homogenizer in 1 ml of cold suspension buffer containing 85 mM sodium chloride (NaCl), 20 mM potassium chloride (KCl), 4 mM magnesium chloride (MgCl), 0.2 mM EGTA and 30 mM histidine pH 7.2. Saponin was added to the samples to a final concentration of 20 μg/ml. They were then incubated at 37°C for 15 minutes. Protein concentration was measured by Bradford assay and concentrations were adjusted to 10 mg/ml.

In a 96-well plate, 60 μl of ATP buffer containing 140 mM NaCl, 20 mM KCl, 3 mM MgCl, 30 mM histidine and 3 mM ATP were added to wells. Two sets of samples were included, one with ATP buffer only and the other with 100 μM of the Na+/K+ ATPase selective inhibitor ouabain added to the ATP buffer. Subsequently, 10 μl of protein homogenates were added to the ATP buffer ± ouabain, which were then mixed by pipetting and incubated at 37°C for 30 minutes. The reaction was stopped by adding 120 μl of an acid molybdate solution consisting of 0.5 g ammonium molybdate (Sigma, St. Louis, MO) in 0.5 M sulfuric acid. After mixing, 10 μl of Fiske Subbarow Reducer (Sigma, St. Louis, MO) was added and wells were mixed again. The plate was allowed to incubate covered at room temperature for 10 minutes and then measured spectrophotometrically at 660 nm. A standard curve of phosphoric acid dilutions was used to calculate the specific activity of the ouabain sensitive ATP hydrolysis and converted to μmols of inorganic phosphate (Pi) liberated/mg protein/ hour. All reactions were performed in triplicate, which were then averaged to produce the single value for the sample. Differences between APP+PS1 and non-transgenic mice were analyzed for significance using one-way ANOVA. Aβ used for these studies was generated by resuspending 1 mg of commercially available recombinant Aβ 1–42 peptide (rPeptide, Athens, GA) in 221 μl of 1,1,1,3,3,3-Hexafluoro-2-propanol (HFIP, Sigma, St. Louis, MO) to generate 45 μg Aβ 1–42 films. These films were resuspended in 2 μl of anhydrous DMSO, followed by agitation and subsequent addition of either 48 μl of cold water followed by overnight incubation at 4°C (for soluble Aβ preparation) or 10 mM HCl followed by overnight incubation at 37°C (for fibrillar Aβ preparation). The acid was neutralized the following day by the addition of NaOH. These preparations yielded approximately 900 μg/ml suspensions of Aβ.

For analysis of activity inhibition by both preparations of Aβ, purified Na+/K+ ATPase (from brain; Sigma, St. Louis, MO) was pre-incubated for 2 hours in a 37°C orbital shaker separately with 112.5, 225 and 450 μg/ml of either a DMSO+water Aβ suspension or a DMSO+neutralized HCl Aβ suspension. Vehicle alone was also used for each preparation and sample values are indicated as a percentage of the "vehicle only" values. Activity was then measured using the same activity assay as above and significance was measured using one-way ANOVA comparing activity between vehicle treated and Aβ treated.

### Western blot

Brain homogenate from cortical tissue was equilibrated to 10 μg of total protein. This homogenate, along with 1 μg of purified cerebral Na+/K+ ATPase, was diluted 1:1 with loading buffer containing 4% SDS and 5% β-mercaptoethanol, heated to 95°C for 5 minutes and loaded onto a 7.5% Tris-glycine gel which was electrophoresed at 100 mV for one hour in the presence of SDS. The protein was subsequently transferred onto an Immobilon membrane (Millipore, Billerica, MA) for one hour at 100 mV. The blot was rinsed with borate saline + 0.05% Tween-20 (BST) and blocked overnight at 4°C in 5% non-fat dry milk (NFDM). The following day, a 1:2000 dilution of rabbit anti-rat Na+/K+ ATPase αIII antibody (Upstate Biotech, Lake Placid, NY) in 0.5% NFDM+BST was applied to the blot for 1 hour, followed by washing and a subsequent 1 hour incubation with a 1:5000 dilution of hrp-labeled anti-rabbit IgG (Sigma, St. Louis, MO) in 0.5% NFDM+BST. After washing, the blot was developed for chemiluminescence using a luminol substrate kit (Santa Cruz Biotech, Santa Cruz, CA). Band density was quantified using the SoftMax Pro program and one-way ANOVA was used to determine significance.

### Histology

Immunohistochemical and immunofluorescence analyses for Na+/K+ ATPase αIII and phosphorylated neurofilament, respectively, were performed on the same 25 μm free-floating hippocampal sections. Sections were treated 15 minutes with 10% methanol, 3% hydrogen peroxide and 80% phosphate buffered saline (PBS) to block endogenous peroxidase activity, and then washed 3 times with PBS. Sections were subsequently treated with sodium borohydride for 15 minutes to reduce background auto-fluorescence [38] followed by washing with PBS. Sections were then permeabilized for 30 minutes with 100 mM lysine, 0.2% triton x-100, 2% goat serum and 2% horse serum in PBS, and washed 3 times with PBS. Because one antibody was murine being used on mouse tissue, endogenous mouse IgG was first blocked with a 1:300 dilution of goat F (ab') 2 anti-mouse IgG (overnight; Protos Immunoresearch, Burlingame, CA). Sections were washed the following day and co-incubated with a 1:5000 dilution of a rabbit anti-rat Na+/K+ ATPase αIII IgG (Upstate Biotech, Lake Placid, NY) and a 1:10,000 dilution of a mouse monoclonal IgG1 ascites pool specific for phosphorylated forms of neurofilament (SMI-312; Sternberger Monoclonals, Lutherville, MD) in 2% goat serum and 2% horse serum in PBS. The following day, sections were washed, and then co-incubated in both a 1:3000 dilution of anti-rabbit biotinylated secondary antibody (Vector Laboratories, Burlingame, CA) and a 1:100 dilution of anti-mouse fluorescein conjugated secondary antibody (Vector Labs, Burlingame, CA) for 2 hours. After washing, the tissue was incubated with Vectastain^® ^Elite^® ^ABC kit (Vector Labs, Burlingame, CA). The tissue was then washed and stained with a diaminobenzidine: peroxide system plus nickel enhancement (DAB/Ni^2+^), followed by final washes. Compact amyloid plaques were visualized using Congo red staining after sections were slide mounted and dried [19]. Briefly, slides were incubated in alkaline alcoholic saturated sodium chloride (AASSC), followed by 0.2% Congo red in AASSC, then dehydrated and cover-slipped with xylene-free Vectamount (Vector Labs, Burlingame, CA). Other mounting media were tested, but only Vectamount was suitable for combined immunohistochemistry, immunofluorescence and Congo red staining. The extent of nonspecific binding was assessed in the absence of primary antibodies for all assays. Specificity of the Na+/K+ ATPase antibody was confirmed by reduced staining following a 2 hour pre-incubation of the antibody with purified cerebral Na+/K+ ATPase at a 1 antibody to 4 enzyme molecule ratio. Both immunostains were characterized individually before the co-incubation procedure was implemented.

## Authors' contributions

C D contributed to the design the study, developed the activity assay, generated the Aβ preparations, performed the histology and western blotting, and drafted the article. M G was responsible for maintenance of the animals used in the study, prepared the tissue for subsequent analysis, and assisted in the design. Both D W and D H participated in the histology and contributed to the coordination of the study. M B facilitated the investigations and assisted with histology. M F performed the genotyping of the mice. D M conceived of the study, and contributed to its design and coordination, along with helping to draft the manuscript. All authors read and approved the final manuscript.
